# Molecular taxonomy of endemic coastal *Ligia* isopods from the Hawaiian Islands: re-description of *L. hawaiensis* and description of seven novel cryptic species

**DOI:** 10.7717/peerj.7531

**Published:** 2019-08-15

**Authors:** Carlos A. Santamaria

**Affiliations:** Biology Faculty, College of Science and Mathematics, University of South Florida, Sarasota, FL, United States of America; Department of Biological Sciences, Sam Houston State University, Huntsville, TX, United States of America

**Keywords:** Oniscidea, Intertidal, Species description, Ligiidae, Pacific biodiversity, Cryptic species

## Abstract

Past phylogeographic work has shown *Ligia hawaiensis,* a coastal isopod species endemic to the Hawaiian Islands, to be a paraphyletic complex of several highly genetically divergent yet morphologically cryptic lineages. Despite the need for a taxonomic revision of this species, the lack of morphological differentiation has proven an impediment to formally describe new *Ligia* species in the region. Molecular characters and species delimitation approaches have been successfully used to formally describe cryptic species in other crustacean taxa, suggesting they may aid taxonomic revisions of *L. hawaiensis*. Herein, various distance- and tree-based molecular species delimitation approaches are applied on a concatenated dataset comprised of both mitochondrial and nuclear gene sequences of *L. hawaiensis* and *L. perkinsi*, a terrestrial species endemic to the Hawaiian archipelago. Results of these analyses informed a taxonomic revision leading to the redescription of *L. hawaiensis* and the description of seven new cryptic species on the basis of molecular characters: *L. dante*, *L. eleluensis*, *L. honu*, *L. kamehameha*, *L. mauinuiensis*, *L. pele*, and* L. rolliensis*. These coastal *Ligia* species from the Hawaiian archipelago appear to be largely limited to single islands, where they appear largely constrained to volcanic rift zones suggesting allopatric events at local scales may drive diversification for poorly dispersing organisms in the Hawaiian coastlines. Additional work remains needed to fully assess the role of said events; however, the description of these novel species underscore their potential to aid in studies of local diversification of marine organisms in Hawai‘i. Lastly, this represents the first application of molecular taxonomic approaches to formally describe genetic lineages found in *Ligia* isopods as species, underscoring the promise these methods hold to taxonomic revisions in other species in the genus shown to harbor cryptic genetic lineages.

## Introduction

The isopod genus *Ligia* (Fabricius 1798) consists of ∼40 currently valid species, most of which inhabit rocky intertidal habitats (Schmalfuss, 2003). The genus is known to exhibit several biological traits that severely limit their dispersal potential: *Ligia* isopods are direct developers who carry their embryos in a brood pouch (i.e., marsupium) until their emergence as fully formed juveniles, have poor desiccation resistance (Barnes, 1934; Barnes, 1935; Tsai, Dai & Chen, 1998), avoid open water (Barnes, 1932), and exhibit poor locomotion outside their rocky habitats (Santamaria personal observation). Such low vagility and the patchiness of *Ligia* habitats (i.e., rocky intertidal coastlines) have been suggested to restrict gene flow, leading to long-term isolation and deep genetic divergence between populations even across small geographic distances (e.g., Jung et al., 2008; Hurtado, Mateos & Santamaria, 2010; Eberl et al., 2013; Santamaria et al., 2013). Not surprisingly, molecular characterizations have uncovered highly divergent genetic lineages in several *Ligia* species around the world suggesting that some species may represent cryptic species complexes in need of formal taxonomic description (Jung et al., 2008; Hurtado, Mateos & Santamaria, 2010; Santamaria et al., 2013; Raupach et al., 2014; Santamaria, Mateos & Hurtado, 2014; Santamaria et al., 2017; Greenan, Griffiths & Santamaria, 2018; Hurtado et al., 2018). One such example is that of *Ligia* from the coastlines of the Hawaiian archipelago.

Two coastal species have been described from the Hawaiian islands to date: *L. hawaiensis* (Dana, 1853) and *L. kauaiensis* (Edmondson, 1931). The former was first described by Dana (1853) from specimens collected in Kaua‘i and O‘ahu and later confirmed as a valid species by Jackson (1922). It is currently thought to occur solely in the Hawaiian archipelago where it is widespread (Taiti & Ferrara, 1991; Taiti & Howarth, 1996; Schmalfuss, 2003). *Ligia kauaiensis* was described by Edmondson (1931) from individuals collected from Kalihiwai Bay, Kaua‘i. He differentiated *L. kauaiensis* from *L. hawaiensis* based on differences in inter-eye distance, number of segments in the flagellum of the antenna, and body size; however, posterior authors did not find such differences and suggested *L. kauaiensis* to be a junior synonym of *L. hawaiensis* (Arcangeli, 1954). The current taxonomy of the *Ligia* genus reflects this synonymy, recognizing *L. hawaiensis* as the sole coastal *Ligia* species endemic to the Hawaiian archipelago (Schmalfuss, 2003). Recent molecular analyses of coastal *Ligia* from the region; however, suggest *L. hawaiensis* may be a cryptic species complex in need of taxonomic revision.

Phylogeographic studies completed in the past two decades suggest *L. hawaiensis* to be a paraphyletic taxon composed of several deeply divergent and cryptic lineages (Taiti et al., 2003; Santamaria et al., 2013). Maximum Parsimony phylogenetic reconstructions carried out in 2003 and based on a mitochondrial dataset including *L. hawaiensis* and *L. perkinsi*, a terrestrial species from the Hawaiian archipelago, from Kaua‘i and O‘ahu uncovered three divergent lineages within *L. hawaiensis* (Taiti et al., 2003). A decade later, Santamaria et al. (2013) expanded on this work by including samples from previously unsampled islands, using additional genetic markers, and applying model-based phylogenetic reconstruction approaches. They found *L. hawaiensis* to be a paraphyletic taxon composed of several highly divergent and geographically disjunct lineages, including a clade comprised of coastal *Ligia* from Maui and Hawai‘i (*Clade A*), another comprised of Kaua‘i individuals (*Clade D*), one containing *Ligia* from O‘ahu and the Maui-Nui complex (*Clade E*), and lastly one comprised of individuals from the islands of O‘ahu, Maui, and Hawai‘i (*Clade F*). Given the paraphyly of *L. hawaiensis* and that levels of divergence between said lineages matched or exceeded those observed between other *Ligia* species pairs, Santamaria et al. (2013) concluded *L. hawaiensis* may represent a cryptic species complex in need of taxonomic revision. Unfortunately, such taxonomic revisions have been hindered by an apparent lack of morphological differentiation amongst highly divergent genetic lineages.

Taiti et al. (2003) evaluated eight characters used in *Ligia* taxonomy for both male and female *L. hawaiensis* of what are now known to be three highly divergent lineages (i.e., *D*, *E*, *F*) and failed to find any differences between them. More recently, geometric-morphometric comparisons capturing characters used in *Ligia* taxonomy (e.g., inter-eye distance, shape of telson) were carried out by Santamaria et al. (2013) to determine whether statistically significant shape differences amongst genetic lineages existed. Although their analyses did recover statistically significant differences amongst lineages, cross-validated discriminant function analyses indicated these to be of no taxonomic value as correct classification rates were as low as 26.67%. Similar results have been since reported for other *Ligia* species. High degrees of overlap in overall body shapes and low classification rates have also been reported for *L. occidentalis* lineages (Santamaria et al., 2016), while taxonomic examination of highly divergent *L. natalensis* lineages uncovered by Greenan, Griffiths & Santamaria (2018) failed to uncover taxonomically diagnostic characters amongst in the lineages found in this southern African species (C.L. Griffiths personal communication). Hence, the totality of these findings indicates molecular approaches may be the best-suited approach to formally describe coastal *Ligia* lineages from the Hawaiian archipelago as species.

Molecular-based species approaches have been successfully used in detecting, delineating, and describing cryptic species in other peracarids such as *Gammarus* amphipods (Grabowski, Wysocka & Mamos, 2017), munnopsid isopods (Schnurr et al., 2018), and *Atlantoscia* isopods (Zimmermann et al., 2018). In this study, phylogenetic reconstructions as well as distance- and phylogeny-based molecular species delimitation methods are applied on a multi-locus dataset comprised of *L. hawaiensis* and *L. perkinsi* individuals collected throughout the Hawaiian archipelago to inform the revision of the taxonomy of *L. hawaiensis*. Results reported herein indicate the need to narrowly re-describe *L. hawaiensis* and to describe seven new species distinguished on the basis of molecular characters. The formal description of these cryptic species not only further highlights *Ligia* as a rare example of *in-situ* speciation in a Hawaiian marine taxon bird (Kay & Palumbi, 1987, but see Bird et al., 2011), but may also be of importance to conservation efforts (Bickford et al., 2007; Delić et al., 2017).

## Materials and Methods

### Sample collection

*Ligia* specimens were collected from 24 rocky intertidal habitats across the Hawaiian islands of Kaua‘i, O‘ahu, Maui, and Hawai‘i in the summer of 2016. Of these, nine are localities previously sampled by Santamaria et al. (2013) with the remaining fifteen being localities previously not characterized by either Santamaria et al. (2013) or Taiti et al. (2003). Detail locality information is provided in Table 1. All individuals were caught by hand during the day and field-preserved in 70% ethanol. Once in the laboratory, all specimens were identified as *L. hawaiensis* by comparing the endopod of the 2nd pleopod to the morphology illustrated by Taiti et al. (2003) prior to molecular characterizations.

**Table 1:** Localities included in the study, with corresponding number of individuals sampled, GenBank accession numbers, and geographic information.

Loc. Label	Locality name	New Loc.	# inds. (New)	COI Acc. No	16S rDNA Acc, No.	12S rDNA. Acc. No.	Cytb Acc. No.	28S rDNA Acc. No.	NaK Acc. No.	H3A Acc. No.	Latitude	Longitude
A1	Wai‘Ōpae Maui	NO	2(1)	MK034488	MK032502 KF546549	MK032601 KF546573	MK034572 KF546718	N/A	N/A	MK034658	20°37′29.20″N	156°12′34.10″W
A2	Kealakukea Bay Hawai‘i	NO	6(5)	MK034474 MK034475 MK034476 MK034477 KF546627	MK032515 MK032516 MK032517 MK032518 MK032519	MK032608 MK032609 KF546574		MK940873 MK940874	KF546594	MK034663	19°28′32.88″N	155°55′11.04″W
A3	Pu’unalu Beach Park Hawai‘i	NO	5(4)	MK034513 MK034514 KF546628	MK032564 MK032565 MK032566 MK032567 KF546551	MK032627 MK032628 KF546576	MK034582 MK034583 KF546716	MK940887 KF546701	KF546593	MK034677	19°08′00.60″N	155°30′18.30″W
A4	Isaac Hale Beach Park Hawai‘i	NO	6(5)	N/A	MK032568 MK032569 MK032570 MK032571 MK032572 KF546550	MK032629 MK032630 KF546575	MK034584 MK034585 KF546717	MK940888 KF546702	MK034645 MK034646 KF546586	MK034678 MK034679	19°27′26.82″N	154°50′31.68″W
A5	Miloli Beach Park Hawai‘i	YES	5(5)	MK034478 MK034479 MK034480 MK034481 MK034482	MK032554 MK032555 MK032556 MK032557 MK032558	MK032623 MK032624	MK034567 MK034568 MK034569	MK940885 MK940886	MK034642 MK034643	MK034675	19°10′58.10″N	155°54′25.10″W
A6	Waianapanapa State Park Maui	YES	5(5)	N/A	MK032492 MK032493 MK032494 MK032495 MK032496	MK032596 MK032597	MK034570 MK034571	MK940866	MK034605	MK034654 MK034655	20°47′21.80″N	156°00′07.90″W
A7	Koki Beach Park Maui	YES	5(5)	MK034483 MK034484 MK034485 MK034486 MK034487	MK032497 MK032498 MK032499 MK032500 MK032501	MK032598 MK032599 MK032600		MK940867	MK034606 MK034607 MK034608 MK034609 MK034610	MK034656 MK034657	20°43′41.62″N	155°59′06.71″W
B1	Nu’uanu Pali O‘ahu	NO	1(0)	KF546661	KF546548	KF546572	KF546719	N/A	N/A	N/A	N/A	N/A
C1	Mt Kahili Kaua‘i	NO	1(0)	KF546660	KF546546	KF546578	N/A	N/A	N/A	N/A	N/A	N/A
C2	Makaleha Mts Kaua‘i	NO	1(0)	KF546659	KF546545	KF546577	KF546723	N/A	N/A	N/A	N/A	N/A
C3	Haupu Range Kaua‘i	NO	1(0)	KF546655	KF546547	KF546579	KF546722	KF546683	KF546592	N/A	N/A	N/A
D1	Kalihiwai Beach Kaua‘i	NO	14(5)	MK034540 MK034541 MK034542 MK034543 MK034544 KF546598 KF546599 KF546600 KF546601 KF546602 KF546603 KF546604 KF546605 KF546606	MK032544 MK032545 MK032546 MK032547 MK032548 KF546544	MK032619 MK032620 KF546571	MK034593 MK034594 KF546721	MK940882 MK940883 KF546686 KF546687 KF546688 KF546689 KF546690	MK034635 MK034636 MK034637 MK034638 MK034639 KF546585	MK034672 MK034673	22°13′05.30″N	159°25′31.15″W
D2	Kauapea Beach Kaua‘i	NO	1(0)	KF546656	KF546543	KF546570	KF546720	N/A	N/A	N/A	N/A	N/A
D6	Hoai Bay Kaua‘i	YES	5(5)	MK034545 MK034546 MK034547 MK034548 MK034549	MK032549 MK032550 MK032551 MK032552 MK032553	MK032621 MK032622	MK034595 MK034596	MK940884	MK034640 MK034641	MK034674	21°52′51.93″N	159°28′25.01″W
E2	Papohaku Beach Park Moloka‘i	NO	1(0)	KF546607	KF546542	KF546569	KF546715	N/A	N/A	N/A	21°10′46.56″N	157°15′5.88″W
E3	North of Puko’o Lana‘i	NO	9(0)	KF546608 KF546609 KF546610 KF546611 KF546612 KF546613 KF546614 KF546615 KF546616	KF546540	KF546565	KF546713	KF546696 KF546697 KF546698 KF546700	KF546587	N/A	21°06′06.84″N	156°45′06.66″W
E4	Manele Bay Moloka‘i	NO	7(0)	KF546643 KF546644 KF546645 KF546646 KF546647 KF546648 KF546649	KF546538	KF546564	N/A	KF546677 KF546678 KF546679 KF546680 KF546681 KF546682	KF546589	N/A	20°44′37.37″N	156°53′12.47″W
E5	Poelua Bay Maui	NO	1(0)	KF546657	KF546532	KF546566	KF546710	N/A	N/A	N/A	N/A	N/A
E6	Spreckelsville Maui	NO	8(0)	KF546595 KF546596 KF546597 KF546650 KF546651 KF546652 KF546653 KF546654	KF546539	KF546567	KF546712	KF546691 KF546692 KF546693 KF546694 KF546695	KF546590	N/A	20°54′31.38″N	156°24′40.26″W
E7	Keanae Maui	NO	6(5)	KF546658	MK032487 MK032488 MK032489 MK032490 MK032491 KF546537	MK032594 MK032595 KF546568	MK034597 MK034598 KF546714	MK940865	N/A	MK034652 MK034653	N/A	N/A
E8	DT Fleming Beach Park Maui	YES	2(2)	MK034550 MK034551	MK032503 MK032504	MK032602 MK032603	MK034599 MK034600	MK940868 MK940869	MK034611 MK034612	MK034659 MK034660	21°00′20.82″N	156°38′58.43″W
E9	Hanakao’o Park Maui	YES	5(5)	MK034552 MK034553 MK034554 MK034555 MK034556	MK032505 MK032506 MK032507 MK032508 MK032509	MK032604 MK032605	MK034601 MK034602	MK940870 MK940871	MK034613 MK034614 MK034615 MK034616	N/A	20°54′34.10″N	156°41′19.03″W
E10	Wawamalu Beach Park O‘ahu	YES	5(5)	MK034557 MK034558 MK034559 MK034560 MK034561	MK032535 MK032536 MK032537 MK032538 MK032534	MK032616 MK032617	MK034603 MK034604	MK940879	MK034628 MK034629 MK034630 MK034631 MK034632	MK034669	21°17′12.51″N	157°40′07.66″W
F1	Pupukea O‘ahu	NO	16(5)	MK034494 MK034495 MK034496 MK034497 KF546617 KF546618 KF546619 KF546620 KF546621 KF546622 KF546623 KF546624 KF546625 KF546626	MK032520 MK032521 MK032522 MK032523 KF546533 KF546531	MK032610 MK032611 KF546562	MK034575 MK034591 KF546709	KF546667 KF546668 KF546669 KF546670 KF546671	MK034621 MK034622 MK034623 KF546591	MK034664 MK034665	21°38′59.70″N	158°03′45.48″W
F2	Pouhala Marsh O‘ahu	NO	1(0)	N/A	KF546532	N/A	KF546710	N/A	N/A	N/A	N/A	N/A
F3	Honomanu Bay Maui	NO	1(0)	N/A	KF546530	KF546563	KF546708	N/A	N/A	N/A	N/A	N/A
F4	Keokea Beach Hawai‘i	NO	1(0)	N/A	KF546529	KF546558	KF546703	N/A	N/A	N/A	N/A	N/A
F5	Onekahakaha Beach Park Hawai‘i	NO	19(5)	MK034520 MK034521 MK034522 MK034523 MK034524 KF546629 KF546630 KF546631 KF546632 KF546633 KF546634 KF546635 KF546636 KF546637 KF546638 KF546639 KF546640 KF546641 KF546642	MK032573 MK032574 MK032575 MK032576 KF546534	MK032631 MK032632 KF546561	MK034588 KF546705	KF546672 KF546673 KF546674 KF546675 KF546676	KF546588	MK034680 MK034681	19°44′16.05″N	155°02′20.15″W
F6	Leleiwi Beach Hawai‘i	NO	1(0)		KF546535	KF546560	KF546706	N/A	N/A	N/A	N/A	N/A
F7	South Point Hawai‘i	NO	6(5)	MK034515 MK034516 MK034517 MK034518 MK034519	MK032559 MK032560 MK032561 MK032562 MK032563 KF546536	MK032625 MK032626 KF546559	MK034586 MK034587 KF546707	N/A	MK034644	MK034676		
F8	Kapa’a State Park Hawai‘i	NO	1(0)		KF546528	KF546557	KF546704	N/A	N/A	N/A	20°12′11.52″N	155°54′6.66″W
F9	Kolekole Beach Park Hawai‘i	YES	5(5)	MK034525 MK034526 MK034527 MK034528 MK034529	MK032577 MK032578 MK032579 MK032580 MK032581	MK032633 MK032634	MK034589 MK034590	MK940891 MK940892	MK034647	N/A	19°52′58.80″N	155°07′07.60″W
F10	Laupahoehoe Beach Park Hawai‘i	YES	5(5)	MK034530 MK034531 MK034532 MK034533 MK034534	MK032582 MK032583 MK032584	MK032635 MK032636	MK034591	MK940893 MK940894 MK940895 MK940896	MK034648	MK034682 MK034683	19°59′36.60″N	155°14′24.01″W
F11	Spencer Beach Park Hawai‘i	YES	5(5)	MK034535 MK034536 MK034537 MK034538 MK034539	MK032585 MK032586 MK032587 MK032588 MK032589	MK032637 MK032638	MK034592	N/A	MK034649 MK034650 MK034651	MK034684 MK034685	20°01′22.41″N	155°49′21.50″W
F12	Baby Beach Maui	YES	7(7)	MK034562 MK034563 MK034564 MK034565 MK034566	MK032482 MK032483 MK032484 MK032485 MK032486	MK032592 MK032593	N/A	MK940864	N/A	N/A	20°54′45.09″N	156°24′16.01″W
F13	Kahaluu O‘ahu	YES	5(5)	MK034489 MK034490 MK034491 MK034492 MK034493	MK032510 MK032511 MK032512 MK032513 MK032514	MK032606 MK032607	MK034573 MK034574	MK940872	MK034617 MK034618 MK034619 MK034620	MK034661 MK034662	21°28′17.81″N	157°50′40.65″W
F14	Kaena Point (North) O‘ahu	YES	5(5)	MK034498 MK034499 MK034500 MK034501 MK034502	MK032524 MK032525 MK032526 MK032527 MK032528	MK032612 MK032613	MK034576 MK034577	MK940875 MK940876	MK034624 MK034625	MK034666 MK034667	21°34′47.46″N	158°14′15.43″W
F15	Kaiaka Bay Beach Park O‘ahu	YES	5(5)	MK034503 MK034504 MK034505 MK034506 MK034507	MK032529 MK032530 MK032531 MK032532 MK032533	MK032614 MK032615	MK034578 MK034579	MK940877 MK940878	MK034626 MK034627	MK034668	21°35′20.62″N	158°07′03.42″W
F16	Kaena Point (South) O‘ahu	YES	5(5)	MK034508 MK034509 MK034510 MK034511 MK034512	MK032590 MK032539 MK032540 MK032541 MK032542 MK032543	MK032618	MK034580 MK034581	MK940880 MK940881	MK034633 MK034634	MK034670 MK034671	21°33′21.21″N	158°14′54.88″W

### Molecular laboratory methods

Total genomic DNA was extracted from pereopods and/or pleopods using the Quick g-DNA MiniPrep Kit (Zymo Research) for 1–5 individuals per locality. Afterwards, four mitochondrial and three nuclear gene fragments were amplified using previously published primers and conditions: (a) a 658-bp segment of the Cytochrome Oxidase I gene (hereafter COI, primers LCO1490/HCO2198; Folmer et al., 1994), (b) a ∼490-bp segment of the 16S rRNA gene (primers 16Sar/16Sbr; Palumbi, 1996), (c) a ∼495-bp segment of the 12S rDNA gene (primers crust-12Sf/crust-12Sr; Podsiadlowski & Bartolomaeus, 2005), (d) a 361-bp fragment of the Cytochrome-b gene (hereafter Cytb, primers 144F/151F and 270R/272R; Merritt et al., 1998), (e) a ∼1,000-bp segment of the 28S rDNA gene (primers 28SA/28SB Whiting, 2002) (f) 664-bp region of the alpha-subunit of the Sodium Potassium ATPase (hereafter NaK, primers NaK-forb/NaK-rev2; Tsang et al., 2008), and (g) a ∼328-bp fragment of the Histone H3 gene (primers H3AF/H3AR; Colgan et al., 1998).

Genomic DNA was also obtained for the syntype of *L. hawaiensis* deposited in the Harvard Museum of Comparative Zoology (MCZ CRU-1543) using a modified version of the protocol of Shokralla, Singer & Hajibabaei (2010): one mL of the specimen’s preservative ethanol was evaporated at 56 °C for 30 min, reconstituted in 250 µL of molecular water, with DNA then extracted using the Quick g-DNA MiniPrep Kit (Zymo Research). Two mitochondrial genes fragments were PCR amplified for this specimen using internal primers designed in Geneious R8.1.9 based on publicly available *Ligia* sequences: a 122-bp fragment of the 16S rDNA gene (16S-LigiaF: 5′-CGCAGTATCCTGACTGTGCT-3′, 16S-LigiaR: 5′-AGCTTTTAGGGTCTTATCGTCCC-3′) and a 212-bp fragment of the COI gene (COI-LigiaF 5′-CTWGGDCAGCCTGGWAGRTTT-3′; COI-LigiaR 5′-MCCTGTTCCTACTCCTCTTTCA-3′). All PCR products were visualized on a 1% agarose gel stained using SYBR-Safe (Invitrogen) prior to sequencing at the University of Arizona Genetics Core (UAGC).

### Sequence alignment and model testing

Sequences produced in this study, with the exception of those for the *L. hawaiensis* syntype, were combined with those produced by Santamaria et al. (2013) and those publicly available in GenBank. The syntype sequences were excluded from the dataset due to their relative short length. Ribosomal genes (16S rDNA, 12S rDNA) were aligned using the MAFFT algorithm (Katoh & Standley, 2013) as implemented in the GUIDANCE2 server (Sela et al., 2015) using standard settings. Poorly aligned positions in these alignments were removed automatically by masking all positions with a confidence alignment score below 1.00. Protein coding genes were aligned using the online MAFFT server (Katoh, Rozewicki & Yamada, 2017) using default settings. No evidence suggestive of pseudo-genes was observed in any of the protein coding genes alignments. Pairwise genetic distances were estimated with the Kimura-2-Parameter (K2P) correction (excluding ambiguous sites) in MEGA v7.0.18 for the COI dataset (Kumar, Stecher & Tamura, 2016).

For each aligned gene dataset, the most appropriate model of nucleotide evolution was selected from 1,624 models by evaluating their likelihoods on a fixed BioNJ-JC tree under the Bayesian Information Criterion (BIC) in jModeltest v2.1 (Darriba et al., 2012). Afterwards, individual gene alignments were concatenated using SequenceMatrix v.1.7.8.1 (Vaidya, Lohman & Meier, 2011) and the most appropriate model of nucleotide evolution selected for the concatenated alignment as described above.

### Molecular species delimitation analyses

Species hypothesis were obtained using several molecular species delimitation analyses (hereafter MSDAs) approaches, including both tree and distance based approaches. Two tree-based MSDA approaches were implemented: the Poisson Tree Processes model used in the PTP server ( http://species.h-its.org/), an approach that delineates species based on branching patterns (Zhang et al., 2013), and the General Mixed Yule Coalescent model (hereafter GMYC; Fujisawa & Barraclough, 2013), an approach that uses branch lengths to determine the transition from intraspecific to interspecific relationships.

As PTP analyses require phylogenetic trees as input, phylogenetic searches on the concatenated alignment of all six genes were carried out under Maximum Likelihood inference as implemented in RAxML v8.0.0 (Stamatakis, 2014). Searches were repeated for three partitioning approaches: unpartitioned, by gene, and as determined by the BIC implemented in PartitionFinder v1.0.0 (Lanfear et al., 2012) using settings as Santamaria et al. (2013). RAxML searches consisted of 1,000 bootstrap replicates followed by a thorough ML search under the GTR + Γ model run under the Thorough Bootstrap Algorithm with all other settings as default. Majority-rule consensus trees were estimated for each analysis using the SumTrees command of DendroPy v3.10.1 (Sukumaran & Holder, 2010). PTP analyses were then carried under both the Maximum Likelihood and Bayesian implementations on both the ML tree and majority consensus bootstrap tree produced by each search. Settings used were as follows: 500,000 MCMC iterations; a burn-in of 0.10; and a thinning value of 100.

In contrast with PTP, GMYC delineations require ultrametric trees as input. Thus, BEAST v2.1.3 (Bouckaert et al., 2014) was used to estimate ultrametric trees for the unpartitioned concatenated mitochondrial dataset using two different approaches: assuming a constant rate of evolution and speciation assuming a Yule process (i.e., constant speciation rate; Yule, 1925; Gernhard, 2008), and under a coalescent model of speciation assuming a constant population size (Kingman, 1982). Both searches were used using the most appropriate model of nucleotide evolution. All BEAST runs were carried out for 50 million generations with sampling every 1,000th. Resulting trees were summarized using the *SumTrees* command with burn-in discarded and with edges set as per the mean-age option. Resulting ultrametric trees were analyzed using the GMYC approach as implemented by the ‘splits’ package (http://r-forge.r-project.org/projects/splits/) in R using default settings.

Two distance-based approaches were applied on the COI gene dataset alone: the ABGD software (Puillandre et al., 2012) and the BIN system applied in BOLD v3 (Ratnasingham & Hebert, 2013). ABGD analyses were carried out on the entire COI dataset and after masking ambiguous sites using the online server (http://wwwabi.snv.jussieu.fr/public/abgd/abgdweb.html) under the Kimura 2-Parameter (K2P) nucleotide evolution model, a *P*_min_ value of 0.01, *P*_max_ of 0.20, and a relative width of 1. All other settings were as default. BIN searches in BOLD were carried out using the “Cluster Sequences” option under the K2P distance model and the BOLD aligner option. Sequences shorter than 200-bp, with evidence of contaminants, possibly misidentified, and with stop codons were filtered out. All other parameters were as default.

Candidate species were then identified by comparing results of phylogenetic reconstructions, pairwise COI K2P distances, and MSDAs patterns. In general terms, candidate species were chosen so that all the following criteria were met: (1) all members of the putative species constituted a well-supported (BS >90%) monophyletic clade recovered in all phylogenetic reconstructions; (2) within-group average pairwise COI K2P distances in the clade were <3.0%; and (3) a majority of MSDAs assigned individuals as belonging to the same candidate species. Exceptions to the third criterion were made for those instances where analyses identified several species consisting of a single individual within a well-supported monophyletic group identified as a single species by other analyses. These criteria were chosen to avoid over-splitting.

Candidate species were validated using BPP v4.0 (Yang, 2015) by completing species delimitations under two different partition schemes (i.e., unpartitioned, by gene) and several combinations of *θ* and *τ* priors. Diagnostic nucleotide positions were then determined for the validated candidate species using the “Diagnostic character” function of BOLD. Analyses were carried out on all genes independently assuming a K2P distance model, all quality filters, grouping of sequences according to species, and alignments as submitted. All other settings were as default. Diagnostic and partially diagnostic characters for each species were recorded, with others ignored.

Lastly, the identity of the name-carrying *L. hawaiensis* syntype deposited in the Harvard Museum of Comparative Zoology was established using three approaches. First, the COI sequence obtained from this specimen was queried against all barcode records available in the Barcode of Life Database (BOLD) in May of 2019 (Ratnasingham & Hebert, 2007). Second, the 16S rDNA and COI sequences were each queried against all published sequences in GenBank using BLAST in May 2019. Lastly, 16S rDNA and COI sequences were combined with the corresponding gene dataset produced in this study, and aligned as described above. Neighbor-joining trees were produced for each aligned gene dataset using Geneious R8.1.9.

The electronic version of this article in Portable Document Format (PDF) will represent a published work according to the International Commission on Zoological Nomenclature (ICZN), and hence the new names contained in the electronic version are effectively published under that Code from the electronic edition alone. This published work and the nomenclatural acts it contains have been registered in ZooBank, the online registration system for the ICZN. The ZooBank LSIDs (Life Science Identifiers) can be resolved and the associated information viewed through any standard web browser by appending the LSID to the prefix http://zoobank.org/. The LSID for this publication is: urn:lsid:zoobank.org:pub:7FF280B9-13B0-43F9-A9D6-1C2CDF29C0CC. The online version of this work is archived and available from the following digital repositories: PeerJ, PubMed Central and CLOCKSS.

## Results

**Figure 1:**
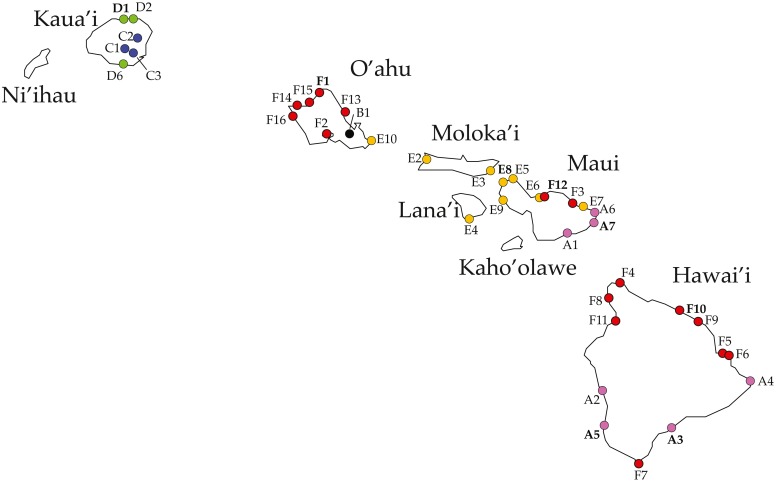
*Ligia* localities included in this study. Labels and colors correspond with other figures and tables in this study and that of Santamaria et al. (2013). Detailed information for each locality is presented in Table 1. Localities of the suppralittoral *L. hawaiensis* included: Kaua‘i: D1-Kalihiwai Beach, D2-Kauapea Beach, D6-Hoai Bay (D6); O‘ahu: E10-Wawamalu Beach Park, F1-Pupukea, F2-Pouhala Marsh, F13-Kahaluu (F13), F14-Kaena Point (North), F15-Kaiaka Bay Beach Park, F16-Kaena Point (South); Moloka‘i: E2-Papohaku Beach Park, E4-Manele Bay; Lana‘i: E3-North of Puko’o; Maui: A1-Wai‘Ōpae; A6-Waianapanapa State Park, A7-Koki Beach Park,E5-Poelua Bay, E6-Spreckelsville, E7-Keanae, E8-DT Fleming Beach Park, E9-Hanakao’o Park, F3-Honomanu Bay, F12-Baby Beach Spreckelsville Area; Hawai‘i: A2-Kealakukea Bay, A3-Pu’unalu Beach Park, A4-Isaac Hale Beach Park, A5-Miloli Beach Park, F4-Keokea Beach, F5-Onekahakaha Beach Park, F6-Leleiwi Beach, F7-South Point, F8-Kapa’a State Park, F9-Kolekole Beach Park, F10-Laupahoehoe Beach Park, F11-Spencer Beach Park. Localities of the terrestrial *L. perkinsi* included are Kaua‘i: C1-Mt Kahili, C2-Makaleha Mts, C3-Haupu Range; O‘ahu: B1-Nu’uanu Pali. Boldfaced labels indicate type localities.

The combination of molecular data produced in this study with that previously published by Santamaria et al. (2013) produced a concatenated dataset 3,889-bp long after to the removal of poorly aligned positions in the 16S, 12S, and 28S rDNA genes (43, 17, and 49 respectively). This alignment included four mitochondrial and three nuclear genes from 193 individuals across 39 localities in the Hawaiian archipelago (Fig. 1, Table 1). The final alignment included 543 parsimony informative sites (COI = 185; Cyt-b: 120; 12S rDNA = 99; 16S rDNA = 91; 28S rDNA = 39; NaK = 6; H3A = 3). All new sequences produced in this study have been deposited in GenBank under accession numbers MK032482 –MK032590, MK032592 –MK032638, MK034474 –MK034685, MK940864 –MK940896 (Table 1) while an annotated alignment is provided as Dataset S1. The sequences produced in this study are also deposited in the BOLD database.

All phylogenetic reconstructions completed in this study were congruent and match those reported by Santamaria et al. (2013). Four highly divergent lineages comprised of coastal *Ligia* were identified: (a) *Clade A* (lavenders and purples in all figures) which contained all individuals from three localities in Maui (A1, 6–7) and Hawai‘i (A2–5) each; (b) *Clade D* (green in all figures) which included all coastal *Ligia* sampled from Kaua‘i (D1–2, 6); (c) *Clade E* (oranges and yellows in all figures) from O‘ahu (E10), Moloka‘i (E2, E3), Lana‘i (E4), and Maui (E5–E9); and lastly (d) *Clade F* (reds in all figures) from O‘ahu (F1–2, 13–16), Maui (F3, 12), and Hawai‘i (F4–F11). No locality was shown to harbor more than one of these lineages. Two highly divergent lineages comprised of *L. perkinsi* individuals were recovered.

**Figure 2:**
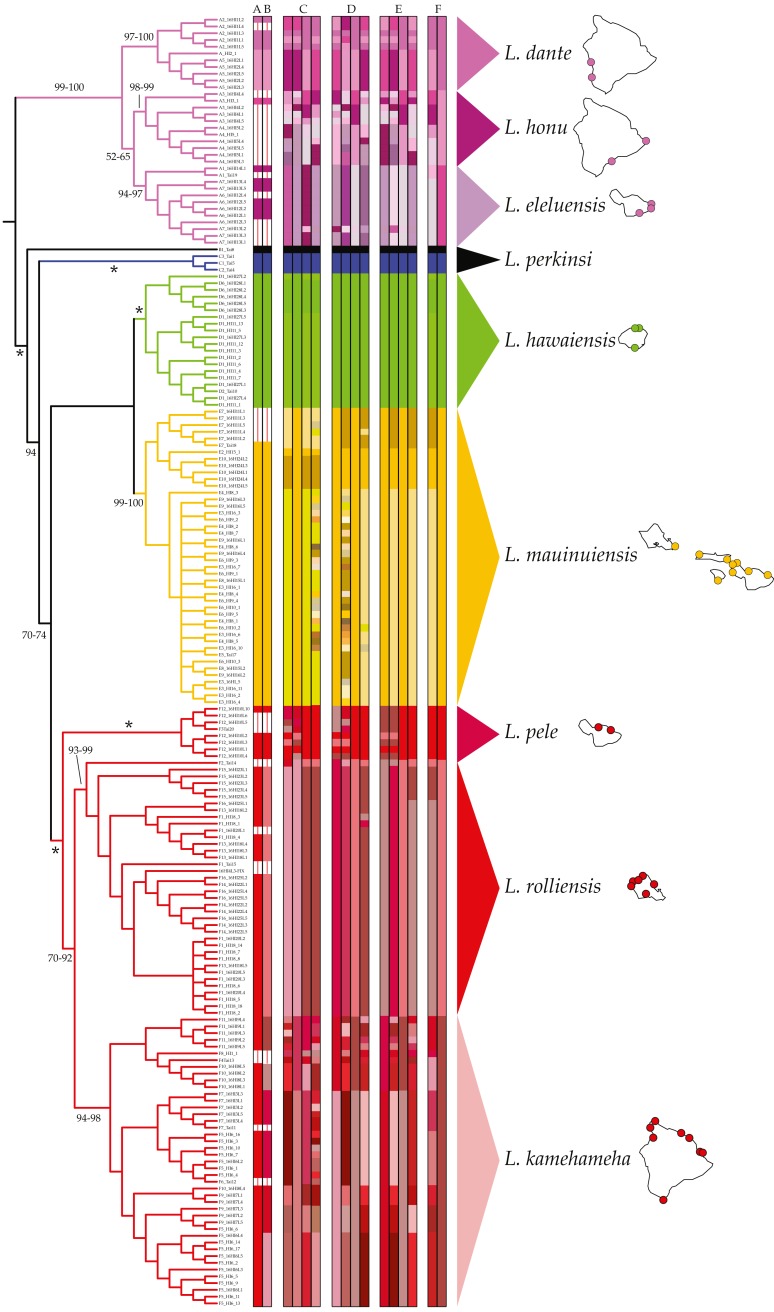
Results of molecular species delimitation analyses (MSDAs). Results are projected on the majority rule consensus tree produced by analyzing the concatenated mitochondrial and nuclear dataset of *Ligia* samples from the Hawaiian Islands in RAxML under the GTR +Γ under a “by gene” partitioning scheme. Branches are transformed for clarity and are colored per clade as per Santamaria et al. (2013). Vertical bars represent assignments to putative species (identified by colors) under various MSDA methods. Values by nodes correspond with bootstrap support values, with * denoting 100% across all analyses. Bars A–B represent assignments by ABGD and BOLD respectively, two distance-based methods on the COI dataset alone. All other bars are results from tree-based approaches. Results for PTP and bPTP (Bayesian implementation of PTP) based on phylogenetic searches carried out on RAxML under various partitioning schemes are presented in C (unpartitioned), D (by gene), and E (according to PartitionFinder). The first two vertical bars within each of these correspond to PTP and bPTP results based on the most likely tree produced by RAxML, with the last two corresponding to PTP and bPTP results on majority consensus bootstrap trees. The bars denoted by F correspond with GMYC assignments based on Coalescent and Yule speciation models respectively. Consensus species as well as the localities where they have been identified at are shown.

MSDAs assigned individuals identified as *L. hawaiensis* to 5–57 putative species. Results across analyses were largely congruent, with higher putative species counts produced by some analyses over-splitting larger groups into species with two or less members. No contradictory assignments (e.g., individuals assigned to different species containing three or more species) were observed. ABGD analyses of the COI dataset identified between 5 and 13 species within *L. hawaiensis*, with 13 and 7 being reported for four partitions each, the former at lower P values (0.010–0.018) and the latter at higher P values (0.022–0.035). BIN analyses in BOLD produced similar results to those of ABGD at higher P values, with the sole difference being the split of *Clade A* individuals into three rather than two putative species. Tree-based MSDAs recognized 8–57 putative species from specimens identified as *L. hawaiensis*, with 3–15 species for *Clade A*, 1–2 for *Clade D*, 1–22 for *Clade E*, and 1–22 for *Clade F*. A detailed breakdown of all MSDA results is presented in Fig. 2.

Comparisons amongst MSDAs, phylogenetic reconstructions, and pairwise COI K2P distances (Table 2) based on the criteria previously led to the identification of eight candidate species of coastal *Ligia* from the Hawaiian archipelago. Three candidate species were identified from *Clade A*: (a) one comprising all individuals collected in localities A2 and A5, (b) one from those collected in A3–4, and (c) another containing all specimens from A1, A6–7. Only one candidate species was identified in *Clade D* (all individuals from localities D1–2, D6) and another in *Clade E* (all individuals from E2–10). Lastly, three candidate species were identified in *Clade F*: (a) one comprised of all individuals collected in Maui (F3, 12), (b) one from O‘ahu specimens (F1–2, F13–16), and (c) another composed of all *Clade F* individuals from Hawai‘i (F4–11). BPP analyses provided high support for this partitioning scheme, as high posterior probabilities (>0.99) were observed for all eight candidate coastal *Ligia* species regardless of priors used. No analysis clustered two or more candidate species with posterior probabilities >0.01.

Of these, the candidate species comprised of *Clade D* individuals (green in all Figures) appears to correspond with Dana’s *L. hawaiensis* description, as BOLD identifications (100% match to a *Clade D* haplotype), BLAST searches (16S rDNA: 98.36% match, 100% coverage; COI: 100% match, 100% coverage), and NJ analyses (not shown) indicate the name-carrying syntype of *L. hawaiensis* to be a member of this candidate species. These results thus indicate the need to narrowly redescribe *L. hawaiensis* and to describe seven novel species of coastal *Ligia* from the Hawaiian archipelago.

**Table 2:** Estimates of evolutionary divergence, as measured by Kimura 2-parameter distances, for *Ligia* species from the Hawaiian Archipelago.

	*L. dante*	*L. eleluensis*	*L. rolliensis*	*L. honu*	*L. kamehameha*	*L. hawaiensis*	*L. mauinuiensis*	*L. pele*	*L. perkinsi*
*L. dante*	0.0–4.6 (2.4)								
*L. eleluensis*	9.7–11.2 (10.7)	0.0–0.9 (0.5)							
*L. rolliensis*	14.0–16.5 (15.2)	13.4–15.4 (14.3)	0.0–2.0 (0.5)						
*L. honu*	5.8–7.5 (6.8)	10.9–11.3 (11.0)	14.9–15.7 (15.2)	N/A					
*L. kamehameha*	13.8–16.4 (14.8)	13.8–15.4 (14.6)	4.0–6.4 (5.0)	13.6–15.5 (14.6)	0.0–5.4 (2.5)				
*L. hawaiensis*	12.8–15.4 (14.4)	14.7–16.4 (15.8)	12.5–14.4 (13.6)	15.0–16.9 (16.4)	11.6–14.6 (13.0)	0.0–2.2 (0.9)			
*L. mauinuiensis*	13.6–15.9 (14.9)	14.4–16.4 (15.4)	10.9–12.6 (11.6)	14.5–15.3 (15.0)	8.7–13.1 (10.6)	10.3–12.7 (11.5)	0.0–2.4 (0.7)		
*L. pele*	15.0–16.6 (15.8)	15.4–16.2 (15.7)	6.1–7.1 (6. 6)	15.9–16.2 (15.0)	6.4–8.7 (7.3)	12.6–14.8 (13.9)	11.1–12.8 (12.0)	0.0–0.2 (0.9)	
*L. perkinsi*	11.9–15.0 (13. 6)	12.5–14.8 (13.6)	12.9–14.7 (13.9)	13.7–15.1 (14.1)	11.1–15.0 (13.2)	13.8–15.8 (15.2)	12.5–16.6 (13.8)	13.9–16.1 (14.9)	1.0–15.3 (8.4)

## Taxonomy

**Figure 3:**
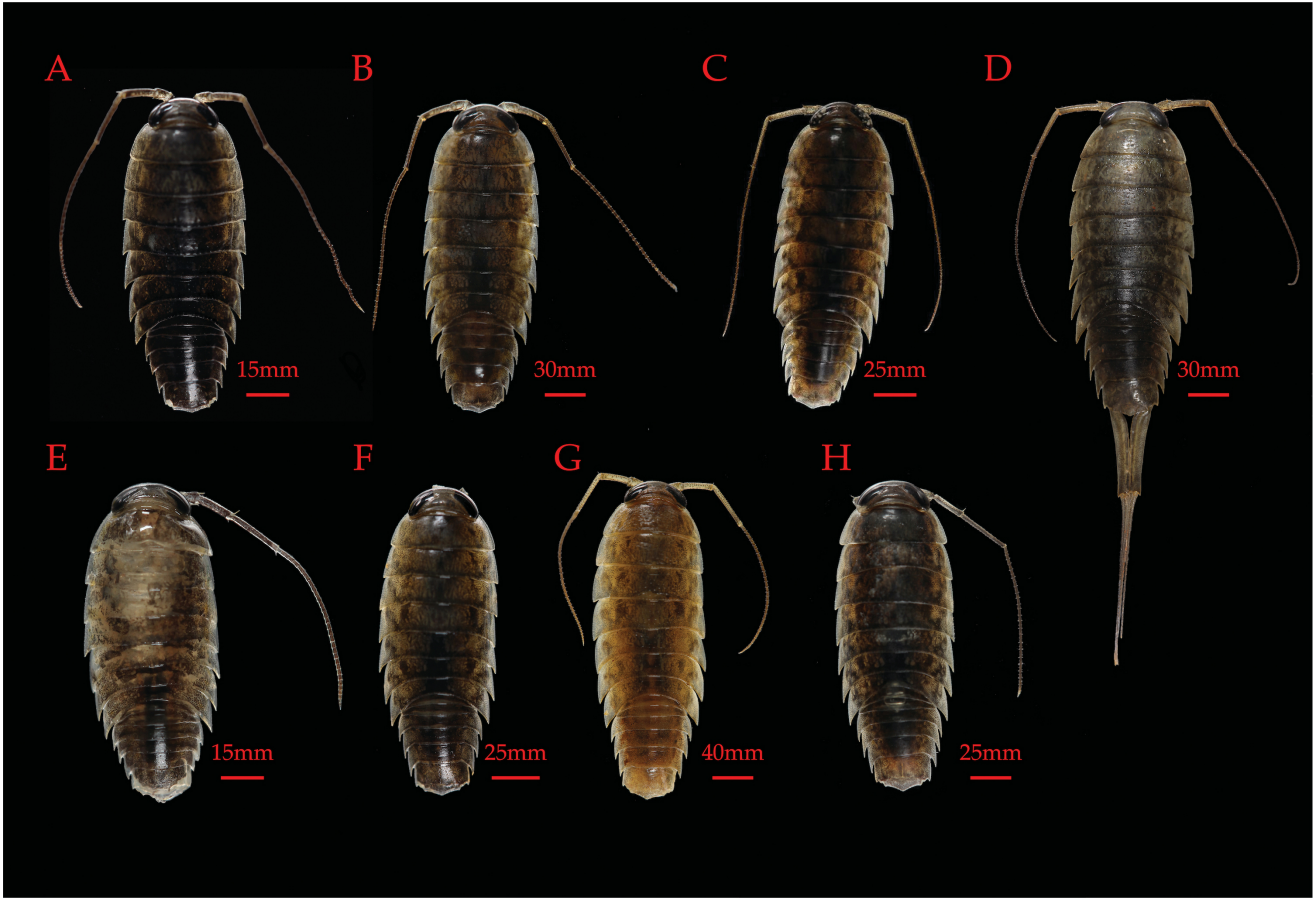
Figure 3: Paratypes of novel and re-described Hawaiian *Ligia* species. (A) *L. honu* (UFID 49319); (B) *L. eleluensis* (UFID: 49329); (C) *L. hawaiensis* (UFID 49341); (D) *L. pele* (UFID 49326); (E) *L. dante* (UFID 49316); (F) *L. kamehameha* (UFID 49322); (G) *L. rolliensis* (UFID 49336); (H) *L. mauinuiensis* (UF 49331).

MSDAs results, as well as those of phylogenetic reconstructions, COI K2P pairwise distances, and the geographic distribution of lineages informed the re-description of *L. hawaiensis* as well as the description of seven novel coastal *Ligia* species from the Hawaiian archipelago. All type specimens, paratypes, and additional lots have been deposited at the Florida Museum of Natural History (FLMNH) in Gainesville, FL, USA. Descriptions below focus on molecular characters, as past morphological inspections have shown *L. hawaiensis* lineages to lack diagnostic morphological differences (Taiti et al., 2003; Santamaria et al., 2013). Nonetheless, descriptions briefly touch on some overall body characteristics that may help distinguish *Ligia* species (e.g., eye size/distance, body ratio) and photographs of all type specimens are provided (Fig. 3). All other traits (e.g., pereopods) are as described and/or illustrated by Taiti et al. (2003), Taiti, Ferrara & Kwon (1992), and Jackson (1933).

**Table:** 

***Ligia hawaiensis*** species re-description
LSID: urn:lsid:marinespecies.org:taxname:257550.
BOLD BINs : AAD0842.

Materials examined: the *L. hawaiensis* syntype deposited in the Harvard Museum of Comparative Zoology (MCZ:IZ:CRU-1543) as well as twenty individuals, both male and female, from 6 coastal localities across the island of Kaua‘i (D1, D2, and 6). A neotype (UFID 49339), paratype (UFID 49341) and a lot of five individuals (UFID 49342) from the type locality as well as five individuals from Hoai Bay (UF 49343) have been deposited.

Type locality: Kalihiwai Beach, Kaua‘i (D1; Lat.: 22°13′05.30″N; Long.: 159°25′31.15″W)

Type: The syntype deposited by Dana appears to be a female in extremely poor condition (i.e., less than half the specimen remains) and with no locality information beyond “Hawaiian Archipelago” available for it. Thus, a neotype has been deposited with the expressed purpose of providing clarity to this particular taxon. The neotype deposited belongs to the lineage as that of Dana’s original syntype and was collected from one of the two islands sampled by said author. It is 17.7 mm long and 6.8 mm wide (body length to width ratio of ∼2.6) has been designated as a neotype. Eyes appear to be moderate in size (eye length is ∼0.5 greatest width of cephalon) and spacing (inter-eye distance ∼0.7 times eye length). Posterolateral processes of the pereionite 7 extend ∼0.6 length of the pleonite 3. Antennae are long, extending almost the entire length of the body. The UFID for the neoype is 49339.

Diagnostic molecular characters:

**Table:** 

*COI:* 108-G; 165-T; 177-C; 279-G; 426-A; 459-G; 492-C; 522-G; 591-C.
*16S:* 6(6)-T; 19(19)-A; 174(186)-C; 224(236)-C; 230(260)-C; 272(302)-A; 306(349)-C; 391(434)-T; 414(457)-T
*Cyt-b:* 115-C; 125-T; 175-A; 250-C.
*12S:* 32(35)-G; 297(297)-G; 318(321)-A; 376(379)-A; 475(492)-C.

Partially diagnostic molecular characters:

**Table:** 

*COI:* 87-G; 90-A; 126-T; 201-C; 300-G; 306-T; 327-G; 393-C; 405-C; 411-T; 417-G; 586-C; 606-G; 706-C.
*16S:* 110(110)-C; 270(300)-A; 273(303)-T; 360(403)-G; 439(482)-C.
*Cyt-b:* 37-G; 109-C; 154-C; 155-C; 187-A; 196-C; 290-G; 295-C; 349-T; 352-C.
*12S:* 33(36)-T; 83(86)-T; 301(304)-G; 319(322)-C.

Distribution: this species appears to be geographically limited to the island of Kaua‘i, where it appears to be the only endemic coastal *Ligia* species. It is widely distributed across the island.

Etymology: The name was originally proposed by Dana to reflect the Hawaiian distribution of this species.

**Table:** 

***Ligia dante*** nov. sp.
LSID: urn:lsid:zoobank.org:act:29042EF5-3FB4-4B34-AE4D-39E9B3462CD4.
BOLD BINs : ADO0227; ACQ3367.

Materials examined: 11 individuals from two localities in the island of Hawai‘i (A2, A5). Both males and females were included. The holotype (UFID 49315), a paratype (UFID 49316), and a lot of five individuals (UFID 49317) from the type locality have been deposited.

Type locality: Miloli’i Beach Park, Hawai‘i, USA (A5; Lat.: 19°10′58.10″N; Long.: 155°54′25.10″W).

Type: small male individual (11.27 mm long) that is 4.40 mm wide at the widest point of the pereionite 4 (body length to width ratio of ∼2.5). Eyes appear to be smaller (eye length is ∼0.4 greatest width of cephalon) and more widely spaced (inter-eye distance ∼1.1 times eye length) than in other *Ligia* in the area. Posterolateral processes of the pereionite 7 extend $\sim \frac{1}{3} $ length of the pleonite 3. Antennae does not extend past pleonites being ∼0.9 of body length. The holotype is deposited in the FLMNH under UFID 49315. GenBank Accession numbers for sequences obtained from the holotype are as follows: MK034481 (COI); MK032557 (16S rDNA); MK032624 (12S rDNA); MK034569 (Cytb); MK034643 (NaK).

Diagnostic molecular characters:

**Table:** 

*COI:* 126-G.
*16S:* 224(236)-G; 291(321)-A.
*Cyt-b: N/A*
*12S:* 9(9)-G.

Partially diagnostic molecular characters:

**Table:** 

*COI:* 123-G; 237-C; 241-C; 259-C; 606-T.
*16S:* 146(158)-A; 269(299)-C.
*Cyt-b:* 55-G; 82-G.
*12S:* 85(88)-C; 105(108)-G.

Distribution: Rocky intertidal habitats in the southwestern region of the island of Hawai‘i: Napo’opo’o Park (A2) and Miloli’i Beach Park (A5).

Etymology: This species is named after Dante Santamaria, a dear friend to the author who recently passed away.

**Table:** 

***Ligia eleluensis*** nov. sp.
LSID: urn:lsid:zoobank.org:act:D9C872EB-8BCD-4B16-8FA7-08B1B805E529.
BOLD BINs : ADO6183.

Materials examined: 12 individuals, both males and females, from three localities in the island of Maui (A1, A6, and A7). The holotype (UFID 49328), a paratype (UFID 49329), and a lot of five individuals (UFID 49330) from the type locality have been deposited.

Type locality: Koki Beach Park, Maui (A7; Lat.: 20°43′41.62″N; Long.: 155°59′06.71″W).

Type: male individual that is 14.98 mm long and 5.41 mm wide at pereionite 4 (body length to width ratio of ∼2.8). Eyes appear to be moderate in size (eye length is ∼0.5 greatest width of cephalon) but somewhat more distant than for most other *Ligia* in the area (inter-eye distance ∼0.8 times eye length). Posterolateral processes of the pereionite 7 extend about $ \frac{1}{3} $ length of the pleonite 3. Antennae does not extend past pleonites and is ∼0.7 of body length. The holotype has been deposited under UFID 49328, with sequences for the holotype found under GenBank Accession numbers: MK034485 (COI); MK032499 (16S rDNA); MK032598 (12S rDNA); MK034608 (NaK); MK034656 (H3A).

Diagnostic molecular characters:

**Table:** 

*COI:* 81-G; 225-T; 324-A; 336-C; 360-G; 456-C; 513-C; 549-C; 696-A.
*16S:* 13(13)-G; 45(45)-G; 168(180)-T; 189(201)-C; 289(319)-C.
*Cyt-b:* 4-C; 22-C; 289-T.
*12S:* 1(1)-C; 19(19)-C; 30(33)-A; 166(169)-A.

Partially diagnostic molecular characters:

**Table:** 

*COI:* 88-C; 129-A; 333-T; 411-G; 504-C; 558-G; 573-T; 663-C.
*16S:* 146(158)-T; 280(310)-G; 291(321)-C.
*Cyt-b:* 52-G; 184-G; 187-G; 257-A; 287-C; 304-A; 307-G; 355-C.
*12S:* 82(85)-G; 85(88)-T; 86(89)-C; 87(90)-T; 382(399)-T.

Distribution: this species has only been identified in three localities in the eastern coastline of Maui: Wai‘Ōpae (A1), Waianapanapa State Park (A6), and Koki Beach Park (A7).

Etymology: *Ligia* isopods are often referred to as “wharf roaches” in common parlance. The proposed species name honors this by incorporating the Hawaiian name for a cockroach (‘elelū) into the species epithet.

**Table:** 

***Ligia honu*** nov. sp.
LSID: urn:lsid:zoobank.org:act:2C6958E3-C573-4F5A-AB97-7AFC19384603.
BOLD BINs : ACQ3366.

Materials examined: 11 *Ligia* individuals, both male and female, collected in two localities in the southern coast of the island of Hawai‘i were examined (A3, A4). The holotype (UFID 49318), a paratype (UFID 49319), and five individuals (UFID 49320) from the type locality have been deposited.

Type locality: Punalu’u Black Sand Beach Park, Hawai‘i, U.S.A. (A3; Lat.: 19° 08′00.60″N; Long.: 155°30′18.30″W).

Type: a 4.97 mm long male individual that is 3.76 mm wide at its widest point (pereionite 4; body length:width ratio of ∼2.6). Eyes appear to be moderate in size (eye length is ∼0.5 greatest width of cephalon) and separation (inter-eye distance is ∼0.7 eye length) when compared to other *Ligia* from the area. Posterolateral processes of the pereionite 7 extend about $ \frac{1}{4} $ length of the pleonite 3. Antennae does not extend past pleonites and is ∼0.7 of body length. The UFID for the holotype is 49318, while GenBank Accession numbers for sequences obtained from this individual are as follows: MK034514 (COI); MK032566 (16S rDNA); MK032627 (12S rDNA); MK034582 (Cytb); MK034677 (H3A).

Diagnostic molecular characters:

**Table:** 

*COI:* 123-A; 126-A; 189-C; 222-C; 234-C; 429-C; 433-C; 597-C; 675-T.
*16S: N/A*
*Cyt-b: N/A*
*12S:* 43(46)-C; 140(143)-G.

Partially diagnostic molecular characters:

**Table:** 

*COI:* 120-T; 321-T; 402-G; 648-C; 678-G.
*16S:* 105(105)-A.
*Cyt-b:* 112-G; 238-G; 281-A; 304-G; 341-T
*12S:* 82(85)-T; 312(315)-C.

Distribution: This species has only been identified in two localities in the southern coastline of Hawai‘i: Punalu’u Black Sand Beach Park (A3) and Isaac Hale Beach Park (A4).

Etymology: The epitet “honu” is derived from the Hawaiian word for turtle and is in reference to the green sea turtles often found resting in the shores of the type locality.

**Table:** 

***Ligia kamehameha*** nov. sp.
LSID: urn:lsid:zoobank.org:act:2EBFFFA4-3BA0-497C-BF97-A09B472893EF.
BOLD BINs : ADN0096; ADN6487; ACQ8239; ACQ8240; ACQ8241.

Materials examined: Forty-three individuals, both males and females, from eight localities in the island of Hawai‘i were examined (F4–11). A holotype (UFID 49321), a paratype (UFID 49322), and a lot of five individuals (UFID 49323) from the type locality as well as five individuals from Onekahakaha Beach Park (UFID 49324) have been deposited.

Type locality: Spencer Beach Park, Hawai‘i (F11; Lat.: 20°01′22.41″N; Long.: 155°49′21.50″W)

Type: a female individual that is ∼2.5 longer than wide at pereionite 4. Eyes are relatively small (∼0.4 times greatest width of cephalon) yet moderately spaced (inter-eye distance ∼0.7 eye length). Posterolateral processes of the pereionite 7 extend to the middle of pleonite 3. Antennae does not extend past pleonites and is ∼0.7 times the body length. The holotype has been deposited under UFID 49321 with sequences available under GenBank Accession numbers: MK034535 (COI); MK032585 (16S rDNA); MK032637 (12S rDNA); MK034592 (Cytb); MK034649 (NaK); MK034684 (H3A).

Diagnostic molecular characters:

**Table:** 

*COI:* 207-A; 243-G.
*16S:* N/A.
*Cyt-b:* N/A.
*12S:* 224(227)-G; 236(239)-C.

Partially diagnostic molecular characters:

**Table:** 

*COI:* 633-G.
*16S:* N/A.
*Cyt-b:* N/A.
*12S:* 352(355)-C.

Distribution: The distributional range of this species appears to be limited to the island of Hawai‘i where it is widespread, particularly across its north and west coasts.

Etymology: The species epithet honors Kamehameha I, founder and first ruler of the Kingdom of Hawaii, who was born in the Kohala region of the island of Hawai‘i where the type location for this species is located.

**Table:** 

***Ligia mauinuiensis*** nov. sp.
LSID: urn:lsid:zoobank.org:act:B8D9EC07-5127-45A4-94D4-8B14C392A2C0.
BOLD BINs : AAD0844.

Materials examined: 40 individuals from ten localities across the islands of Maui (E5–9), Moloka‘i (E2, E4), Lana‘i (E3), and O‘ahu (E10). A holotype (UFID 49344) from the type locality as well as a paratype (UFID 49331) and a lot of five individuals (UFID 49332) from Hanakao’o Park have been deposited.

Type locality: DT Fleming Beach Park, Maui (E8; Lat.: 21°00′20.82″N; Long.: 156°38′58.43″W)

Type: male individual that is ∼2.8 times longer than wide with average sized and spaced eyes (eye length is ∼0.5 greatest width of cephalon, inter-eye distance ∼0.7 eye length). Posterolateral processes of the pereionite 7 extend about $ \frac{1}{3} $ the length of the pleonite 3. Antennae does not extend past pleonites and is ∼0.70 of body length. Body is finely granular. The holotype has been deposited under UFID 49344, with sequences obtained from this individual available under GenBank Accession numbers: MK034550 (COI); MK032503 (16S rDNA); MK032602 (12S rDNA); MK034599 (Cytb); MK034611 (NaK); MK034659 (H3A).

Diagnostic molecular characters:

**Table:** 

*COI:* 99-G; 303-G; 450-C; 535-T; 564-T; 567-C; 609-G.
*16S:* 93(93)-T; 143(143)-A; 258(288)-C; 335(378)-T.
*Cyt-b:* 283-G; 340-A; 346-G.
*12S:* 139(142)-G; 246(249)-A; 262(265)-G; 306(309)-G; 349(352)-A.

Partially diagnostic molecular characters:

**Table:** 

*COI:* 120-G; 318-T; 429-T; 525-A; 631-T.
*16S:* 235(265)-G; 294(324)-C; 440(483)-T.
*Cyt-b:* 181-T; 217-T; 223-T; 275-A; 292-C; 301-C; 304-C.
*12S:* 148(151)-A; 199(202)-T.

Distribution: This species appears to be widespread across the islands of the Maui-Nui group as well as the eastern coastlines of O‘ahu.

Etymology: The species name proposed reflects the distribution of this species primarily across the islands of the Maui-Nui group.

**Table:** 

***Ligia pele*** nov. sp.
LSID: urn:lsid:zoobank.org:act:9E02F410-21B0-4D86-92D3-1CC4E3FBAF05.
BOLD BINs : ADN2023.

Materials examined: eight individuals from two localities in north Maui were examined (A6, A7). The holotype (UFID 49325), a paratype (UFID 49326) and a lot of five individuals (UFID 49327) from the type locality have been deposited.

Type locality: Baby Beach, Spreckelsville, Maui (F12; Lat.: 20°54′45.09″N; Long.: 156°24′16.01″W).

Type: male specimen that is 14.95 mm long and 5.69 mm wide at its widest point (pereionite 4; body length to width ratio of ∼2.6). Eyes appear smaller than other *Ligia* from the area (eye length is ∼0.4 greatest width of cephalon) and wide set (inter-eye distance is equal to eye length). Posterolateral processes of the pereionite 7 extend more than $ \frac{3}{4} $ length of the pleonite 3. Antennae does not extend past pleonites and is ∼0.8 body length. The holotype is deposited under UFID 49325. Sequences produced from this individual are available under GenBank Accession numbers: MK034562 (COI); MK032482 (16S rDNA).

Diagnostic molecular characters:

**Table:** 

*COI:* 228-C; 234-A; 369-T; 462-G; 474-C; 564-G.
*16S:* 127(127)-G; 340(383)-T; 355(398)-G.
*Cyt-b: N/A*
*12S:* 13(13)-G; 207(210)-G.

Partially diagnostic molecular characters:

**Table:** 

*COI:* 43-T; 81-A; 141-A; 318-C; 519-C; 681-G.
*16S:* 11(11)-G; 102(102)-G;
*Cyt-b: N/A*
*12S:* 223(226)-G; 305(308)-G; 474(491)-C.

Distribution: This species has been recorded in two localities in northern Maui: Honomanu Bay (F3) and Baby Beach in Spreckelsville (F12).

Etymology: The name of this species honors the Hawaiian deity Pele.

**Table:** 

***Ligia rolliensis*** nov. sp
LSID: urn:lsid:zoobank.org:act:760DB916-8442-42B8-A6E5-09E32773001A
BOLD BINs : AAD0843.

Materials examined: 37 individuals, both males and females, from six localities across O‘ahu were examined (F1, F2, F13–16). A holotype (UFID 49334), paratype (UFID 49336) and a lot of five individuals (UFID 49337) from the type locality as well as an additional five individuals from Kaena Point (North; UFID 49338) have been deposited.

Type locality: Pupukea, O‘ahu (F1; Lat.: 21°38′59.70″N; Long.: 158°03′45.48″W).

Type: This specimen is a male that is 21.50 mm long and 8.18 mm wide at its widest point (pereionite 4) resulting in a body length to body width ratio of ∼2.6. Eyes appear to be of moderate size when compared to other *Ligia* from the area (eye length is ∼0.4 greatest width of cephalon) with a distance between the eyes that also appears comparable to most other *Ligia* in the region (inter-eye distance is ∼0.7 of eye length). Posterolateral processes of the pereionite 7 extend about halfway of the length of the pleonite 3. Antennae does not extend past pleonites and is ∼0.7 body length. The neotype is deposited under UFID 49334. GenBank Accession numbers for the neotype specimen are as follows: MK034494 (COI); MK032521 (16S rDNA); MK032611 (12S rDNA); MK034622 (NaK); MK034665 (H3A).

Molecular diagnostic characters:

**Table:** 

*COI:* 520-T.
*16S:* N/A.
*Cyt-b:* 302-A.
*12S:* N/A.

Partially diagnostic molecular characters:

**Table:** 

*COI:* 579-T; 672-C.
*16S:* N/A.
*Cyt-b:* 40-C; 325-C.
*12S:* 225(228)-G; 351(354)-G.

Distribution: Widespread across the island of O‘ahu, excluding the eastern tip of the island.

Etymology: This species reflects both a term commonly used for terrestrial isopods in the U.S.A (i.e., rollie pollies) and that of a beloved pet who recently passed away: Rollie.

## Discussion

Phylogeographic work on *Ligia* from the Hawaiian archipelago has uncovered several genetically divergent yet morphologically cryptic lineages in *L. hawaiensis*, suggesting this coastal species endemic to the region represents a cryptic species complex (Taiti et al., 2003; Santamaria et al., 2013). Results of MSDAs implemented on a molecular dataset comprised of mitochondrial and nuclear markers lend further support to this idea, as 5–57 putative species were identified from *L. hawaiensis* individuals, further underscoring the need for a taxonomic revision of the *Ligia* from Hawaii. Ideally, such taxonomic revisions would entail an integrative approach incorporating several lines of evidence (e.g., morphology, mDNA, nDNA; Schlick-Steiner et al., 2010); however, morphological variation appears to be severely limited across genetically divergent *L. hawaiensis* lineages (Taiti et al., 2003; Santamaria et al., 2013). Similarly, variation in nuclear markers appears to be limited perhaps as a result of the young age of the *Ligia* lineages in the region (Santamaria et al., 2013). Thus, species descriptions in this study relied primarily on mitochondrial data, a suitable approach for delineating cryptic species and/or organisms with a young evolutionary history (Schlick-Steiner et al., 2010; Jörger & Schrödl, 2013; Grabowski, Wysocka & Mamos, 2017).

In the case of coastal Hawaiian *Ligia*, results from MSDAs, phylogenetic patterns, distributional data, and molecular diagnostic characters analyses led to the re-description of *L. hawaiensis* and the description of seven novel species in the area. *Ligia hawaiensis* is herein re-described to represent the sole coastal *Ligia* lineage found in the island of Kaua‘i, as both COI and 16S rDNA sequences obtained from the syntype of *L. hawaiensis* was identified as a member of *Clade D* (greens in all figures). This finding cements the status of *L. kauiensis* as a junior synonym of *L. hawaiensis*, as *Clade D* also includes *Ligia* individuals from the type locality of *L. kauaiensis* (Kalihiwai Bay, Kaua‘i). It also underlines the potential of non-destructive approaches in producing molecular data from historical specimens, as the DNA obtained for the >150 year old syntype was extracted from its fixative using a variant of the protocol proposed by Shokralla, Singer & Hajibabaei (2010).

The seven novel species described mainly appear to be allopatric species that are primarily found in the younger Hawaiian islands. Three novel species are found solely on the island of Hawai‘i: *L. dante* from the South Kona district in the south-west, *L. honu* in the Ka‘u and Puna districts along the south and southeastern coast, and *L. kamehameha* in the coastlines of the Kohala, Hamakua, and Hilo districts. Another two newly described species exhibit geographic ranges limited to the island of Maui: *L. pele* from the north of the island, and *L. eleluensis* from its eastern coastline. Lastly, *L. mauinuiensis* is described from localities in the islands of Maui, Lana‘i, Moloka‘i, and the eastern coastline of O‘ahu, while *L. rollie* is found solely in O‘ahu. These general patterns suggest that most of the *Ligia* species in the Hawaiian archipelago may be relatively young species (most species found in islands <2 myo; (Carson & Clague, 1995)) largely confined to a single island where they exhibit disjunct geographical distributions, and may thus be informative on the processes driving diversification for coastal organisms across local scales in Hawai‘i.

Although additional work remains needed to fully delineate the distributional limits for these new species, particularly in islands where more than one species is found (e.g., O‘ahu, Hawai‘i), distributional patterns observed to date suggest local-scale allopatric events (e.g., volcanic or hydrogeographic processes) may have driven the diversification of *Ligia* populations at local scales. For instance, *Ligia* species in the island of Hawai‘i exhibit distributional breaks that partially match volcanic rift zones: *L. dante* appears to be distributed solely within the western coast line of the Mauna Loa rift zone, *L. honu* within the Kilauea rift zone, and *L. kamehameha* primarily found in the Mauna Kea and Kohala rift zones (see Fiske, Jackson & Sutton, 1972). Similar phylogeographic patterns have been observed for *Halocaridina rubra* (Craft et al., 2008) and vermetid snails (Faucci, 2007) in the island of Hawai‘i, suggesting that allopatric processes associated with volcanic rift zones may have played a role in the diversification of other poorly dispersing coastal organisms in the Hawaiian island, even across small distances. Additional sampling and the application of genomic approaches may not only further elucidate the role of these allopatric events but also whether any introgression amongst species is occurring.

Highly divergent genetic lineages representing possible cryptic species have been reported from several *Ligia* species (Jung et al., 2008; Hurtado, Mateos & Santamaria, 2010; Eberl et al., 2013; Raupach et al., 2014; Santamaria, Mateos & Hurtado, 2014; Santamaria et al., 2017; Greenan, Griffiths & Santamaria, 2018; Hurtado et al., 2018); however, this represents the first attempt at formally describing such lineages as species. Findings of this study underscore the value molecular taxonomic approaches hold for completing taxonomic revisions in this isopod genus. Thus, their application in taxonomic evaluations of other *Ligia* species known to harbor highly divergent lineages is recommended. Doing so may help clarify the taxonomy of these isopods while updating it to match the levels of genetic diversity that have been reported so far in these species and may aid in the conservation of coastal biodiversity across the globe.

## Conclusion

Phylogenetic reconstructions combined with molecular species delimitation analyses using both mitochondrial and nuclear gene fragments led to the re-description of *L. hawaiensis* and the description of seven novel coastal species in the area. These species are largely allopatric, exhibiting non-overlapping geographic ranges in the Hawaiian Archipelago. As such, they appear to represent another case of local diversification in a Hawaiian marine organism. Further studies of these organisms may thus be informative on the processes responsible for diversification in Hawaiian coastlines. Lastly, the successful application of MSDAs in this study suggest these approaches may be suitable to formally describe cryptic species in this genus in other regions of the world where high levels of genetic divergence have been reported from.

##  Supplemental Information

10.7717/peerj.7531/supp-1 Dataset S1 Concatenated alignment used for phylogenetic analyses and MSDAs

10.7717/peerj.7531/supp-2 Supplemental Information 1 Neighbor-Joining tree of COI haplotypes. Colored taxon labels represent those used in all other figures

## Additional Information and Declarations

### DNA Deposition

The following information was supplied regarding the deposition of DNA sequences: All new sequences produced in this study are available in GenBank: MK032482 –MK032590, MK032592 –MK032638, and MK034474 –MK034685.

### Data Availability

The following information was supplied regarding data availability: An annotated version of the alignment used in analyses is available as a Supplemental File.

### New Species Registration

The following information was supplied regarding the registration of a newly described species: Publication LISD: urn:lsid:zoobank.org:pub:7FF280B9-13B0-43F9-A9D6-1C2CDF29C0CC *Ligia dante* LSID: urn:lsid:zoobank.org:act:29042EF5-3FB4-4B34-AE4D-39E9B3462CD4 *Ligia honu* LSID: urn:lsid:zoobank.org:act:2C6958E3-C573-4F5A-AB97-7AFC19384603 *Ligia eleluensis* LSID: urn:lsid:zoobank.org:act:D9C872EB-8BCD-4B16-8FA7-08B1B805E529 *Ligia mauinuiensis* LSID: urn:lsid:zoobank.org:act:B8D9EC07-5127-45A4-94D4-8B14C392A2C0 *Ligia kamehameha* LSID: urn:lsid:zoobank.org:act:2EBFFFA4-3BA0-497C-BF97-A09B472893EF *Ligia pele* LSID: urn:lsid:zoobank.org:act:9E02F410-21B0-4D86-92D3-1CC4E3FBAF05 *Ligia hawaiensis* LSID: urn:lsid:marinespecies.org:taxname:257550 *Ligia rolliensis* nov. sp LSID: urn:lsid:zoobank.org:act:760DB916-8442-42B8-A6E5-09E32773001A.
