# Supplementary figures and images for: Molecular taxonomy of endemic coastal Ligia isopods from the Hawaiian Islands: re-description of L. hawaiensis and description of seven novel cryptic species
Source: PeerJ. 2019 Aug 15;7:e7531. doi: 10.7717/peerj.7531 (PMC6698373; doi:10.7717/peerj.7531)

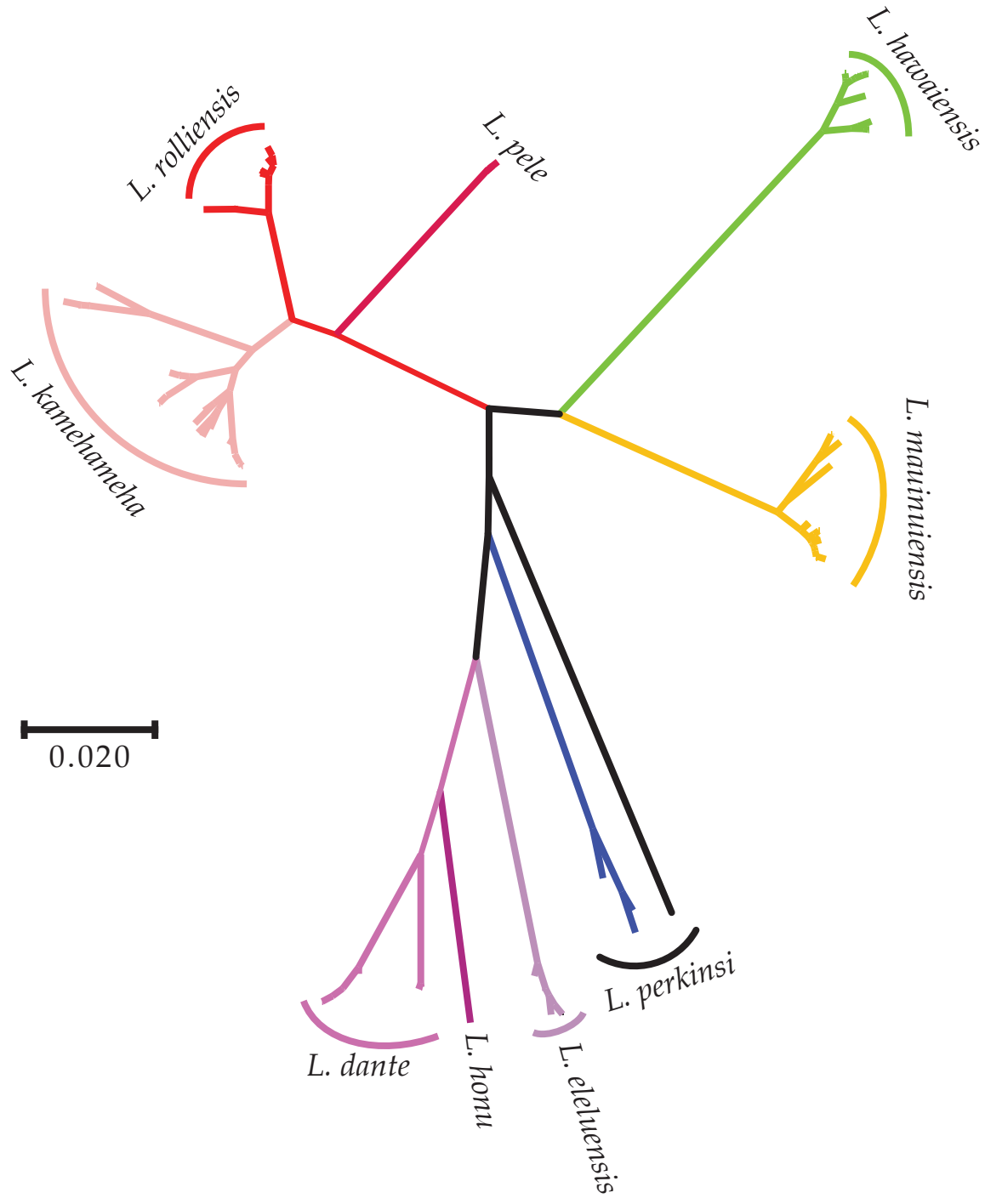

Supplement: Supplemental Information 1 [file peerj-07-7531-s002.pdf]
